# Embedding Physical Therapy in the Pediatric Primary Care Setting: Qualitative Analysis of Pediatricians’ Insights on Potential Collaborative Roles and Benefits

**DOI:** 10.3390/pediatric16040073

**Published:** 2024-10-09

**Authors:** Ryan P. Jacobson, Rebecca R. Dobler

**Affiliations:** Doctor of Physical Therapy Program, George Fox University, 414 N. Meridian St., Newberg, OR 97132, USA; rdobler@georgefox.edu

**Keywords:** thematic analysis, medical home, value-based healthcare, upstream care, rehabilitation

## Abstract

The growing need for collaborative healthcare teams to meet complex health challenges has led to physical therapists (PTs) being embedded in adult primary care settings for many years now. However, this model of care has not been found in pediatrics. This qualitative study sought to gain insights from pediatricians on the potential of embedding pediatric PTs in primary care. Participants were nine pediatricians practicing in both urban and rural, hospital-based and private settings. Semi-structured interviews were recorded, transcribed, and analyzed via thematic analysis per published methods, assuring trustworthiness. Three overarching themes emerged: pediatricians’ priorities aligned with the Quadruple Aim of Healthcare, embedded PTs could fill multiple roles in pediatrics, and they could see a wide variety of patients, highlighting real potential benefits in primary care. Participants endorsed in-office focused treatments, screening to determine optimal care pathways, and ongoing patient follow-up as potential PT roles in this setting. Providers thought that PTs could help manage care for musculoskeletal complaints, high-risk infants, medically complex children, autism, and obesity. An advanced-trained PT having attributes of confidence, adaptability, and open-mindedness was desired. All participants endorsed pediatric primary care PTs as having potentially high value in their practice. This is the first known study on the potential of embedding a PT in the pediatric primary care setting, offering valuable insights from pediatricians to be leveraged in implementation planning.

## 1. Introduction

There is an increasing need for interdisciplinary healthcare teams to meet community needs around mental health, non-communicable disease, persistent pain, and musculoskeletal complaints [1,2]. Primary care providers are on the front lines in addressing this increasing challenge. The Agency for Health Care Research and Quality identifies integrated health centered within the patient-centered medical home (PCMH) as the way of the future, toward healthcare that is proactive rather than reactive [3,4]. The PCMH comprises five aspects for delivery of holistic care, including a comprehensive care model (e.g., a team of clinicians), patient-centered care (i.e., patients and families are at the center of decision making), coordinated care, accessible services (e.g., shorter wait times, 24 h access), and quality and safety (e.g., evidence-based practice, outcome measurement, patient satisfaction) [3]. This PCMH model of care is well supported in the literature for both adult and pediatric care, including integration of psychology, pharmacy, social work, and dietetics [5,6,7]. As primary care is not defined by the credentialed providers on the team but rather the skill sets needed to optimally support a population, there is also interest in embedding physical therapists (PTs) as a part of the PCMH team [8]. With an ever-decreasing amount of primary care providers [9] and increasing challenge in managing non-communicable disease [10], effective management and care continuity for adult and pediatric patients is a real challenge. The need for other disciplines to provide interdisciplinary collaborative care is paramount for improving patient outcomes, curbing medical costs, and retaining providers [11]. A meaningful portion of such care can be effectively delivered in the PCMH by PTs, whose scope and expertise are well suited to providing integrative and lifestyle care management in that setting.

In the military in the United States, PTs have been embedded in primary care settings since the Vietnam War [12]. Privileges extended to military PTs extend beyond treating neuromusculoskeletal (NMSK) problems to include ordering imaging and laboratory tests, limited pharmacologic prescription, and specific injections, requiring additional training with recertification occurring every two years [12]. In this military model, PTs have performed beyond their civilian peers in management of NMSK conditions via conservative care, doing so upstream in the continuum of primary care (i.e., at point of patient entry into the care system), with no or minimal adverse events reported. Physicians’ challenges around providing optimal and timely care have been lessened, as the collaborative work of these PTs has contributed to decreased patient visits, decreased chronicity of injuries, and triage of acute issues with referral to the appropriate provider [13].

Indeed, realities in healthcare exist that explain the emergence of embedded physical therapy in primary care practices. McKay et al., 2021 reported that 20–25% of all primary care visits are NMSK-related [14]. Cieza et al., 2021 reported that with increasing population age and rates of non-communicable diseases, it is estimated that one-third of individuals worldwide would benefit from preventative and proactive access to rehabilitation [15]. In this systematic analysis, Cieza et al., 2021 employed a stepwise analysis and convened an expert panel through World Health Organization to identify 25 health conditions contributing toward the most years lived with disability. For all ages, conditions such as NMSK (62% increase in prevalence, globally, between 1990 and 2019), sensory impairments (77%), cerebral palsy (50%), neurological disorders (106%), chronic respiratory conditions (89%), and mental disorders (40%, including developmental intellectual disabilities) were identified as significant contributors to global disease burden that could be greatly impacted by rehabilitation experts in a primary care setting. This global increase in prevalence, including in children, might be mitigated with quicker identification in primary care including earlier intervention to decrease severity and improve quality of life across the lifespan [15]. There is a call for more integrated collaborative care in the pediatric PCMH, and pediatric PTs might offer significant value in such a setting.

Despite the apparent need, this model of embedded physical therapy in upstream primary care pediatrics does not appear to exist anywhere in the world. There are examples of downstream specialty practice outside of the United States where embedded pediatric physiotherapy is being employed. In Australia, Canada, the United Kingdom, Norway, and New Zealand, advanced practice physiotherapists (APPs) provide direct-access collaborative care for conservative musculoskeletal management in orthopedics [16]. In Ireland, APPs have decreased orthopedic surgeons’ workload by 10–15% over the course of six years [17]. At a Canadian gait clinic, 96% of orthopedic patients were completely managed by the APP [18]. For children with cerebral palsy, APP-led specialty clinics exist in Sweden and Australia focusing on hip stability over time. These services are co-located and have led to a 62% decrease in wait time for new visits and follow-up appointments with the orthopedic surgeon [19]. In a similar service, outcomes for club foot treatment showed no difference between physiotherapists and orthopedic surgeons in a prospective study of 185 feet [20]. These specialty practice APP examples provide evidence that pediatric PTs with advanced training are able to appropriately and safely manage patients with a wide range of orthopedic conditions, potentially to great benefit upstream in pediatric primary care.

One well-established model for appraising value in health care is the Quadruple Aim, which seeks improved patient experience and outcomes, decreased costs, and enhanced clinician well-being, leading to increased health equity [2]. This has likewise been a useful framework for implementation of adult primary care physical therapy in civilian settings [12,21,22], helping to frame primary care needs, opportunities, and considerations. When considering the potential of a novel pediatric primary care physical therapy service, understanding pediatrician priorities framed withing these Quadruple Aims seems a great place to start. These are the front-line providers managing the ever-increasing complexity of needs in the children and families they serve. Hence, their insights around the on-the-ground realities of pediatric primary care must be understood when considering the potential of embedding a pediatric PT in the pediatric PCMH. Therefore, the purpose of this exploratory study was to describe themes and insights identified by pediatricians on the potential of embedding a pediatric PT within the pediatric primary care setting.

## 2. Materials and Methods

### 2.1. Research Design

This IRB-approved qualitative study (George Fox University IRB Approval #2222011) was conducted using semi-structured interviews (SSIs) and thematic analysis, employing an inductive analysis approach to divulge themes. The researchers sought to explore the thoughts of pediatricians on the potential of embedding a pediatric PT within the pediatric primary care setting. A purposeful sample of pediatricians (n = 9) practicing primary care in both rural and urban geographical areas and representing both private practices and hospital-based entities in western Oregon, USA, was used.

Sample size was determined based on thematic saturation of the data. All participants were recruited via snowball sampling, including word of mouth. Sampling in qualitative analysis cannot be determined a priori. In the context of varying opinions about determining qualitative sample size, thematic analysis and saturation was conceptualized as the standard for the inductive analysis approach used [23]. As this was an exploratory study to determine pediatricians’ thoughts and concerns around the embedding of physical therapy in primary care, the depth of the data produced was considered in determining saturation [24].

### 2.2. Materials and Procedures

All SSI sessions were conducted by the primary investigator (RJ) as per published guidelines [25] in order to ensure consistent administration and an efficient participant experience. These occurred in-person, individually, or in small groups as per provider preference, in a private setting to maintain confidentiality between February and April 2023. The primary investigator was a board-certified pediatric physical therapist and tenured professor highly invested in research around the potential of implementing a primary-care-embedded physical therapy model. An interview schedule was first reviewed and revised by a PhD research faculty colleague trained in qualitative research methodology who was otherwise not involved in the study. This schedule of questions was adhered to, with 11 primary question stems (always asked in the SSI) and related probing questions (asked as needed to further bring forth participant ideas regarding the primary question stem) (Appendix A). Questions were intended to bring out providers’ individual perspectives on the potential of having a PT embedded in the pediatric primary care office, using an “open” interview style that provided enough structure to guide the conversation and determine similar patterns [25]. In each interview, the researcher was allowed to diverge from the interview schedule in order to gain further participant insights, as long as this remained in line with the pre-defined domain of inquiry and categories of the topic (Appendix A). Audio recordings of all SSIs were collected using a hand-held recording device and verified for subsequent transcription.

Prior to beginning the interview, informed consent was obtained from each participant. A brief written description of the study was provided and basic demographics were then collected (Appendix B). After responding to the first four question stems in the interview schedule, participants viewed a short video describing primary care physical therapy (YouTube link: https://youtu.be/Rw5JN-cD1ak, accessed 27 September 2024). The purpose of the video was to explain how embedded PT works in adult primary care, give real-world examples, and briefly describe potential benefits of an embedded pediatric PT. Showing the video after the first four question stems was intentional, allowing providers to first offer their unfiltered thoughts around their own practice experience. Following the video, the researcher answered any questions from participants related to the video and/or primary care PT. Then, the discussion continued as per the interview schedule.

### 2.3. Trustworthiness of Data

Trustworthiness (validity) in qualitative research is established with use of the appropriate methodology for the specific inquiry. In this study, inductive analysis was applied as appropriate for inquiries where there is not much known about the phenomenon under consideration. Purposive sampling was employed as integral to the analysis, in order to gather the information necessary to answer the research question. Validity was further established by performing constant comparative analysis in code generation (credibility) and the other elements essential for trustworthiness in qualitative research. Transferability and dependability were ensured via a rich description of the data, especially through direct quotes, and well-described data collection techniques and methodology. Confirmability was ensured via member checking and triangulation [23].

Upon completion of each interview, audio recordings were transcribed (with all identifying information omitted) and verified by graduate student researchers, each trained in all study procedures by the primary investigator. Thematic analysis of each written transcript was conducted manually by the researchers through extensive iterative read-throughs, coding, and theming, as per Nowell et al., 2017. More specifically, this included familiarization with each transcript, initial general coding of all comments, identification of inter-related codes, pre-theming, peer debriefing, iterative formalization of emergent primary themes and subthemes, and ongoing reference back to the transcripts [23]. A coding journal was kept documenting all analysis steps and theming iterations for the thematic analysis. All recordings, transcribed data, and theming work was kept secure in password-protected electronic files and/or locked filing cabinets, with only the research team having access to these.

Thematic analysis commenced following the first interview session, continuing as additional participants completed interviews. Recruitment of participants was concluded when it was deemed that theme saturation was reached. Specific steps for thematic analysis and triangulation of emerging themes were completed by the primary investigator and the graduate student researchers as follows:

1. Two student researchers (SC and CM) and the primary investigator separately read and re-read the first transcript, familiarizing themselves with its content while recording reflective thoughts on potential pre-theming and connections between ideas;
2. The two student researchers then formally coded the first transcript independently, defining initial themes. These were then added to the shared coding journal;
3. The primary investigator then facilitated a peer debriefing session with SC and CM where coding and initial themes were triangulated, along with discussion on thought processes and the framework used during analysis of the first transcript. Areas of congruence were identified and discussed, and differences were reconciled toward a common approach moving forward in thematic analysis. Based on this discussion, SC and CM returned to their respective transcripts and made adjustments and/or notations based on this peer debriefing;
4. All remaining transcripts were analyzed by only one student researcher (either SC or CM). Subsequent peer debriefing between SC, CM, and the primary investigator occurred regularly. Evolving theme/subtheme information was added to the coding journal as each transcript analysis was completed;
5. Next, SC, CM, and the primary investigator each separately diagrammed a model for themes and subthemes. This group met again to triangulate data and theming, from original transcripts, journal theming, and independently developed diagramming;
6. At this point, in order to decrease the risk of bias in the emerging themes and subthemes, the four additional researchers separately engaged with the transcripts alongside the three initial diagrams developed by SC, CM, and the primary investigator. Each of these four researchers had transcribed at least one audio recording and had familiarity with those specific transcript(s);
7. All researchers independently re-engaged with the materials, seeking to determine (1) important themes and subthemes, (2) impactful insights from providers, and (3) key quotes exemplifying these;
8. Final peer debriefing and triangulation amongst the research team was facilitated by the primary researcher. When consensus was reached, the primary investigator aggregated the team’s collective input into themes and subthemes agreed upon;
9. One pediatrician participant was willing to review our results (member checking), affirming 100% of the resulting themes and subthemes presented here.

## 3. Results

Participant and practice characteristics of the pediatricians interviewed in this study are described in Table 1, comprising seven MDs (five pediatric fellows) and two DOs, having 6 to 33 years’ experience in pediatric practice. Primary care practice settings represented were mainly urban (67%) but roughly equivalent in hospital-based (45%) versus private practice (55%). Participants reported that the patients served most frequently were infants/toddlers, followed by preschoolers, with overall patient loads categorized most often as general medical, followed by mental health. Developmental delay was reported by only two pediatricians as representing “a good amount” of their caseload, and musculoskeletal conditions by only one.

**Figure 1:**
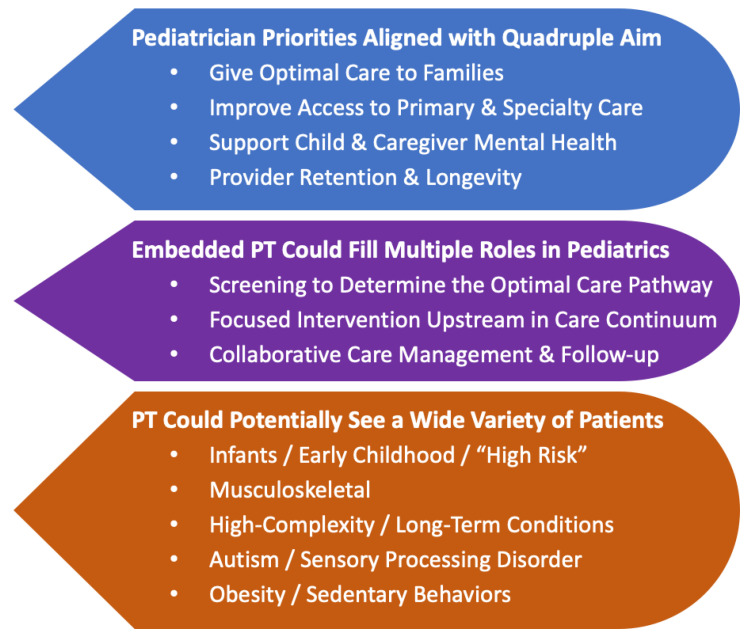
Three themes and related subthemes derived from pediatrician interviews, describing priorities and potential roles for an embedded physical therapist (PT).

Interview sessions lasted an average of 44 min (26 to 59) with all primary question stems asked and responded to 100% of the time. Interestingly, only three pediatricians expressed familiarity with the Quadruple Aim in Healthcare [2], though all endorsed it as being in alignment with their own practice priorities. In addition to responding to questions and probes, all nine participants asked questions to better understand how an embedded primary care PT service might work, and all endorsed support for a potential pilot implementation. Figure 1 reflects three overarching themes derived from the thematic analysis, each with multiple subthemes as follows.

### 3.1. Theme #1: Provider Priorities Aligned Well with the Quadruple Aim

Aligning well with multiple components of the Quadruple Aim, four priority subthemes emerged as participants discussed their own motivations in practice, their perceived effectiveness, ongoing challenges, and the potential of embedded PT. These priorities were: Give Optimal Care to Families; Improved Access to Primary & Specialty Care; Support Child & Caregiver Mental Health; and Provider Retention & Longevity.

All providers interviewed endorsed a priority to Give Optimal Care to Families, though also expressing frustration with the difficulty of doing so consistently in their primary care environment. Aspects of optimal care that emerged most often included having enough time to address the needs and concerns of the child and family in the visit, obtaining a good diagnosis to guide next steps, assuring continuity of care, and ensuring that families feel heard.

“*I mean good pediatric practice unfortunately to me sometimes feels more like luck, and that’s not a very good feeling cause we’re in a scientific field…but the things that you help with are trying to listen well, trying to hear what’s going on, trying to observe well, try to do good physical exam.*”

[Participant 2]

“*Primary is connecting well with the kids and their parents. Without that, nothing else happens.*”

[Participant 8]

“*The thing that would enhance it [practice] is how you get from what you’ve done in the room to having something that follows that patient or that family—that they feel like they’re being cared for and that whatever recommendation you’re making can funnel into their daily life… making sure they understood what was going on and getting to wherever you were referring them.*”

[Participant 7]

All participants expressed the intention that each patient should reach the right place or specialist with the appropriate expertise, efficiently and with assured subsequent follow-up. Additionally, many providers suggested that it was important to minimize families’ added cost burden for primary-care-embedded services. Eight out of nine providers had experience utilizing embedded services, including behavioral health, dieticians, and even one speech–language pathologist. All supported the value of working side-by-side with these clinicians to meet immediate patient needs in their office.

Relatedly, participants were all concerned with finding paths to Improved Access to Primary & Specialty Care for families. This subtheme informed discussions around interview questions related to leading challenges, the participant’s sense of competence and effectiveness, and how embedded PT might better support patient care. Interestingly, some pediatricians in this study reported having sought additional informal training from specialist colleagues to help meet their particular patient population needs. This included training in acute sports medicine rehab, basic behavioral health techniques, safe prescription of additional psychiatric medications, and managing infant torticollis.

“*So I’m spending time talking about how they’re going to rehab from their ankle injury or whatever…You know we talked about everybody working at the top of their license, that somebody else that knows how to do those things could teach them, but I don’t have somebody in that position.*”

[Participant 8]

“*I think were routinely prescribing medicines that are out of our comfort zone—all kinds of stuff I wasn’t prescribing before starting this job, but working with a psychiatrist and getting comfortable with these medicines. We normally wouldn’t routinely prescribe them, but if we don’t do it no one is going to do it.*”

[Participant 4]

“*True, if I could just do the well checks and the preventative care—all the sick visits—that would be great. Some days I just leave the office frustrated. If I wanted to be a psychiatrist, I would have done that degree. But we have to be.*”

[Participant 4]

When asked about priority types of patients that are most difficulty to manage in primary care, participants unanimously endorsed the challenge to effectively Support Child & Caregiver Mental Health. Common needs described ranged from a substantial increase in child and/or parent anxiety over the past few years to complex, multi-factorial mental health conditions for which there are very limited behavioral health resources in the community, in rural and urban contexts alike.

“*Those are probably my parents that have their own mental health, substance abuse, age or socioeconomic factors…the care I am able to give or can’t give even down to just like anticipatory guidance is one of the biggest determinates of how that is going to go for me in that relationship.*”

[Participant 3]

“*Absolutely, and then also just like not even that severe for me and my patient panel. It’s just the highly anxious parents…those families just tend to need more time.*”

[Participant 1]

“*Just the burden on either patients with mental health issues or the parents that have challenges, and lacks of access to services and for services in the community. I think providers are just feeling pretty helpless with, you know, kind of where parents are at.*”

[Participant 9]

Amidst interview discussions around optimal care, access, and challenging aspects of primary care practice, most pediatricians expressed the priority of Provider Retention & Longevity. When asked what personal traits and behaviors are important for a pediatrician’s sense of competence and effectiveness, the most commonly endorsed attributes were patience and “playing the long game” with families, not trying to solve it all, not “going it solo” but leaning on colleagues, and having boundaries that still allow for relatedness and trust with families.

“*Yeah, I think the biggest challenge is just the need, and wanting so desperately to meet it and meet it well, but also having to deal with the fact that we [providers] are finite—both at work and that we all have responsibilities at home as well. And trying to figure out any way of doing that well is just gut wrenching, and I feel personally that often what is lost is any sense of being a person. So what does my patient need? What does my clinic need? What does my family need? And that’s more than the hours in the day.*”

[Participant 3]

### 3.2. Theme #2: Embedded Physical Therapy Could Fill Multiple Roles in Primary Care Pediatrics

Based on a broad range of input from pediatrician participants, three central subthemes capture the potential roles a PT might fill in the primary care pediatric office: Screening to Determine Optimal Care Pathway; Focused Intervention Upstream in the Care Continuum; and Collaborative Care Management & Follow-up.

Affirming the priority of having patients get the right intervention or referral efficiently, participants broadly supported embedded PT having a role in Screening to Determine the Optimal Care Pathway for each patient. This was widely endorsed in particular for patients seeking primary care for a specific problem and/or when an additional concern had arisen beyond the chief complaint. It was affirmed that PT could add much value in collaboratively determining “next steps” for care across a wide range of diagnoses and concerns (see Theme 3). One participant also suggested that the PT could first examine a child or adolescent when the chief complaint was acute injury or a musculoskeletal complaint. Based on presentation and screening of other systems, the PT would then determine whether or not a provider would also need to be called in. Two other participants proposed that the PT might screen appropriate patients first if the provider were running late.

“*I think what you could say is, ‘Hey Dr. [name] is running a little late seeing another patient. I was going to come in and see if you had any questions about the musculoskeletal system?*”

[Participant 3]

Having the PT facilitate preventative health screening visits was also proposed, though providers expressed hesitancy around such things as valuable to families in improving outcomes, as well as families already having too much on their plate and even not showing up.

“*I was thinking that it would…be a cool piece to have in a peds office. A [preventative] visit like that could be a different specialty all together, doing their own assessments. They could catch things early that then [problems] could be prevented—and teaching. I don’t know, I wonder if there’s a place for that somewhere.*”

[Participant 2]

“*The only thing I would worry about with PT and preventative is patients being like, ‘I didn’t really need that.’ You know?*”

[Participant 1]

“*I see it working much better if it’s a needs-based [appointment], as opposed to preventative only, because they’re motivated to stay. And they’re so overwhelmed. I mean you see those mamas at two weeks and one month, and they’re looking at you, they haven’t slept, they can’t even take my anticipatory guidance. All of a sudden you put in a PT you don’t know and I just worry it’s going to push them over the edge.*”

[Participant 2]

Having PTs involved in routine well-child checks to compliment preventative screening was discussed in multiple interviews. Pediatricians expressed potential value for certain children or ages, in particular NICU graduates, children beyond infant age, and developmental/musculoskeletal screening. However, pediatricians noted that the well-child check is an invaluable opportunity for them to connect with families, fostered over time.

“*I could see very much giving over pieces of the well child check, I mean in terms of the development of the musculoskeletal sort of stuff, so totally. I think there is such a—I mean it’s what keeps people in primary care is the longevity of relationship, so we tend to not want to give any of that over because that’s the value, right? That I have seen you. You see somebody, and they say she [the pediatrician) is the first person outside of your dad to hold you, you know? So I think it would be hard to give that up…So we wouldn’t want to lose that, but I think coming alongside somebody who has expertise, who is better at something than I am—absolutely that’s what I want for my patients.*”

[Participant 3]

“*I don’t know if I could let go of that control, ‘cause I want to know I’m seeing them at the 2 month, and the 4 month, and the 6 month, and the 9 month, and the 12 month.*”

[Participant 2]

“*This weekend I had a kiddo come out of the NICU [with] a normal MRI, normal EEG, looked good…But if that was my patient and we had an embedded PT, I’d just automatically flag that kid to be seen by you [PT] at 1 month and at 2 months. And I would just tell parents I’m going to be part of it and PT’s is going to be part of it, you know?*”

[Participant 1]

Participants also endorsed the potential value of in-office Focused Intervention Upstream in the Care Continuum by the PT, though there was limited understanding expressed around how that might specifically look. Many of the providers had previous experience with “warm handoffs” and “brief interventions” provided in the exam room by embedded behavioral health practitioners, expressing their general support for PTs functioning similarly in primary care. One specific insight from various participants was that offering embedded PT might mean those patients who would not typically choose to go to therapy could receive at least one valuable exposure to PT benefits with actionable takeaway points and handouts.

“*I think if we could start doing it in the room—that you could give them a handout that was high quality and some of the techniques to do—and maybe the patient would be more likely to start [outpatient PT], right.*”

[Participant 6]

“*But even to just give families like three things to try at home and follow up in 2 weeks to see if it’s getting better.*”

[Participant 3]

“*You [pediatrician] could do three warm handoffs in the time you could do a brief intervention…I would prefer if they could do the brief intervention…it would be delightful.*”

[Participant 1]

Two specific ideas expanding on potential PT intervention models within the primary care office were each offered by at least two participants, based on past experiences with embedded behavioral health. One was a “50/50 model” whereby the PT would be available half the time for warm handoffs, the other half for scheduled PT-only appointments. The other was for the PT to provide two to three “bridge appointments” for patients originally seen for a warm handoff and then referred to an outpatient PT, as providers described frustration with long wait times for outpatient therapy.

Participants actively engaged interview questions around the potential of embedded PT, identifying many ways it might contribute in Collaborative Care Management & Follow-up. Pediatricians suggested that a PT could increase the frequency of patient monitoring in the primary care office for certain conditions, while also offering improved accessibility for families to attend in-office PT follow-up. This would be possible through such factors as the familiar healthcare location and appointment process of the primary care office, the continuity of having the same PT from the initial “warm handoff” also providing the follow-up, more flexible availability of PT appointments, and even stacking subsequent PT visits before or after the family’s primary care appointment.

“*Anything that can be timely, evidence-based, patient problem focused…I mean I think is always going to make things better for the patient. And I think eliminating those barriers of 17 steps.*”

[Participant 5]

“*I think that’s very streamlined to a follow-up plan. I think one of the things…that behavioral health works so well is that they come down, meet them, do kind of the ice breaker, and automatically say ‘I will follow up—you’re going to follow up with me in 2 weeks in this office right here.’ So I think that also has been very key.*”

[Participant 4]

“*I think this would be a huge value to anyone who has a bunch of NICU patients in their practice—you know, for those kids in that ‘cuspy area’…at their 4 month well child visit. And if the [screening] is worrisome, then you make sure that they get to the NICU follow-up clinic, and if the [screening] is fine, then maybe you…follow them a little more closely.*”

[Participant 7]

“*Having someone who can really carry out the plan and then having that person be able to help the family with whatever was problematic, that piece is the biggest piece to me in terms of like day to day. You want to be done with this patient so you can clear your head and move onto the next patient and give them everything you want to give to them.*”

[Participant 7]

Relatedly, many providers identified the potential value in consulting with PT to determine the best specialist and/or testing referral for certain patients, also endorsing the potential role of the embedded PT in assuring follow-through by the referred family.

“*Certainly, some of the guidance around injury management things, or things that might take time. You [PT] might be able to keep those [appointments] shorter. You might be able to do a follow-up visit for some of that, and then decide if we need more medical evaluation... So that coordination might go better or be more effective in a primary care home, rather than having them go to another place.*”

[Participant 9]

“*I had a mom come in and…she wanted an x-ray, and she asked what they thought, and I was like honestly we would get the x-ray to make her feel better. It would be super useful because parents want it, but saying a physical therapist doesn’t think there’s a need, they might reconsider.*”

[Participant 5]

“*I think as far as this is concerned, you want to send them somewhere but then we get a letter a month later saying the patient did not follow through. And the number one thing I see is PT—I send them to PT then I get a letter later saying the patient declined services. In the room they sound good—‘I’m excited to do it!’…but PT more often than not it seems like they never go to even their first appointment.*”

[Participant 6]

Frequently expressing their difficulty in ensuring that some families follow through on the recommended care plan, pediatricians proposed ways that embedded PT might augment family buy-in. Proposed ideas included the following: (1) more families might value going to an outpatient PT following a warm handoff where the embedded PT has just provided immediate relief/benefit to the child; (2) family anxiety might be decreased, via both the added time spent in the examination by the PT and/or having “another voice” to assure the family when things are fine; and (3) families might be more likely to follow through when a second professional affirms the provider’s diagnosis and prognosis.

“*It allows you to really show the family that degree of concern. And especially if they’re minimizing something, I’m going to have the physical therapist come in. We’re going to look at this together —‘I can see that you’re worried. I think this is going to be OK, but I’m going to have our physical therapist come in. We’re going to take a look together. Let’s get another set of eyes and hands-on here, and let’s make sure that we’re giving you the best answer we can.*’”

[Participant 7]

The value of collaborative care was also proposed to include reciprocal training, such that pediatricians and PTs working side-by-side might build clinician effectiveness. Many participants endorsed the idea that working with other professions could elevate their own skill level in that specialist’s area of practice.

“*Absolutely. I think from a, you know, provider satisfaction [perspective] to watch how a physical therapist assesses—that helps me learn. How are you [PT] looking at that knee or that shoulder? What questions did you ask? What did you recommend? And so I think that, from my standpoint… it’s like a little mini CE [continuing education].*”

[Participant 7]

“*I think for me having time to educate myself on how I could do things better… or how I could recommend certain types of PT activities to the patient myself. Yeah, like with ortho. I’m horrible at ortho personally.*”

[Participant 4]

**Figure 2:**
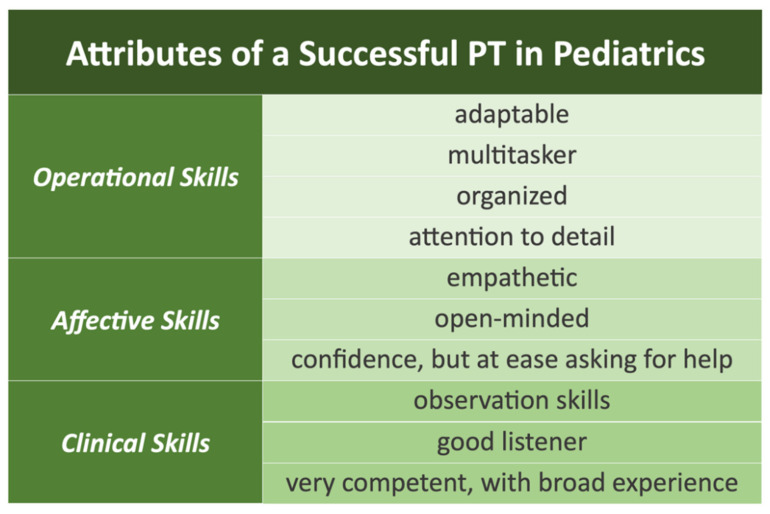
Attributes identified by pediatricians for a successful physical therapist (PT) working in a pediatric primary care setting.

It was also proposed that the embedded PT would become better equipped to screen for important medical signs and symptoms, providing a second pair of eyes and ears after the pediatrician leaves the examination room in a warm handoff. It was also endorsed that the warm handoff and after-visit briefing between pediatrician and PT instills confidence in each other’s skill set. Most participants did state that an embedded PT filling the various roles in primary care described above would need certain attributes to be successful. Figure 2 shows the specific words and phrases describing such a PT. Multiple pediatricians envisioned such a clinician to be someone with many years of experience in pediatrics.

### 3.3. Theme #3: Physical Therapy Could Potentially See a Wide Variety of Patients

As pediatricians considered the potential for PT embedded in primary care, five categories of patients were identified by at least two participants as potentially appropriate for collaborative care management in this setting. These are described below, along with any specific conditions highlighted by one or more interview participants.

With eight out of nine pediatricians identifying infants and toddlers as the population they saw most regularly, most indeed agreed the PT could have an important role in the area of infant/early childhood/“high-risk” populations. High-risk infants included NICU graduates and those with early movement deficits predictive of likely future neurological diagnoses (e.g., cerebral palsy). One provider stated “multiple infant things” might be appropriate for PT, with many participants endorsing the value of calling in a PT for suspected developmental delay and any infant musculoskeletal concerns.

All participants supported the central role of PT in the care of musculoskeletal conditions from birth through adolescence. Specific types of patients more commonly discussed included infants with torticollis and/or plagiocephaly, children with pain (including those with no specific injury), and those with sports injury (including concussion). Management of acute injury was included, as frustration with wait times for outpatient PT was expressed.

Another general category of patients identified were those with high-complexity/long-term conditions. Though agreeing that these comprised a small percentage of infants and children seen in primary care, participants expressed the importance of collaborating with PTs when managing children with complex physical challenges (also including those with chronic and/or multisystem disease). These cases would include diagnoses such as cerebral palsy or other movement disorders.

The two final categories identified here represented conditions that many participants were unsure whether the PT would have a role in managing. Long-term diagnoses of autism/sensory processing disorder were discussed by two different pediatricians—considering whether an experienced PT might be able to contribute to decision making in relation to their care—describing these as high-frequency developmental concerns seen in primary care pediatrics. Perhaps viewed more within the scope of the PT was childhood obesity/sedentary behavior, with some pediatricians especially interested in the potential role of PTs in activity promotion and preventative education. There was positive dialogue with apparent willingness to explore the potential of PTs in both these categories.

## 4. Discussion

This study is the first known investigation into the potential for embedding PTs within pediatric primary care practice. Thematic analysis revealed potentially impactful insights from pediatricians. Related to pediatric practice, substantive challenges to providing optimal care for children and families were identified, with pediatricians feeling frustrated around limited visit times, inconsistent continuity of care and follow-up, and lack of patient access to specialty care (e.g., mental health) and community resources. These are likely to contribute to the increasing burnout rates amongst primary care providers in the United States [9], yet these are factors that might be improved by an embedding a PT well trained in upstream management of a variety of pediatric conditions. Indeed, participants endorsed embedded physical therapy for more than just focused in-office treatment. Screening to determine an optimal care pathway and collaborative care with ongoing follow-up were also identified as potentially valuable PT roles in the pediatric primary care setting. Relatedly, participants endorsed many types of conditions that an embedded PT might see beyond just musculoskeletal diagnoses, including high-risk infants, patients who are medically complex, autism, and obesity. Collaborative support by embedded PTs for pediatricians across a broader range of conditions (beyond musculoskeletal) would be targeted toward improving outcomes across all four goals of the Quadruple Aim [2,17]. Such a role would probably require an advanced-trained pediatric PT with some years of prior experience [17,26,27], which multiple participants in our study endorsed when describing the necessary professional attributes of the PT. Ultimately, there was very positive support from participants, with all nine providers seeing embedded PT in pediatric primary care as potentially high-value and confirming that they would be interested in pursuing the possibility of implementation in their practice.

The pediatricians in this study are managing an increasingly complex caseload, as has been seen across primary care in the US even prior to the COVID-19 epidemic [9,28]. Commitment to obtaining a good diagnosis, continuity of follow-up care, and ensuring families are really heard—particularly in relation to mental health needs—were resounding priorities expressed, aligning with what has been reported in the literature [29,30]. However, it was evident in most interviews that achieving these priorities is incredibly taxing on pediatric providers. It is likely that provider retention and longevity are often realized through significant personal sacrifice and/or compensation [9]—for example, a few participants reported uptraining in behavioral health techniques and safe prescription of additional psychiatric medications. It is evident from the interviews that a key underlying systems challenge is the paucity of time to complete the work, both in the examination room and in ancillary care management activities. Could a well-trained embedded pediatric PT help with these challenges?

Outside of the United States, APPs with specialty training now perform key evaluation and treatment functions in pediatric orthopedics, such as ordering imaging, administering joint injections, and determining whether children should be evaluated for surgery [18,26,27]. Though a “downstream” service in the healthcare continuum, these APPs train specifically with their surgeon counterparts to fulfill their collaborative role effectively [17,27]. Pediatricians in our study noted that they would prefer an embedded physical therapist to have expertise in a wide variety of conditions, helping to ensure that patients are seen by the right professional sooner. Indeed, in pediatric APP specialty services, multiple substantive benefits have been documented—screening, decreased wait times to see specialist physicians, increased percentages of patients who actually need a surgeon filling those surgeons’ schedules, and preventative guidance by the APP for “normal variations” in musculoskeletal presentation [17,19,26,27]. There has also been expansion of APP practice for children into rheumatology clinics, neurology, pain management, and emergency departments [16]. However, other than in emergency care, these APPs are not accessed at the point of patient entry into the healthcare system. Based on our findings, it is likely that pediatric primary care practices would see multiple tangible upstream benefits—for providers and patients—by adding an embedded PT.

Despite the expressed challenges, it was very evident that pediatricians are consistently thoughtful stewards of the Quadruple Aim [2] in their own day-to-day practice. The subthemes Give Optimal Care to Families, Improve Access to Primary & Specialty Care, and Support Child & Caregiver Mental Health all align with the aims of improving the patient experience, improving outcomes, and decreasing costs. For example, in the adult primary care setting, embedded PTs currently provide initial physical therapy services and home exercise programs to bridge care until the patient is able to access outpatient services and/or other specialty care downstream [21]. In infants and younger children with musculoskeletal, developmental, or neurological conditions, earlier intervention has been shown to lead to better long-term outcomes [19,31,32,33,34,35]. Embedded PTs can also meet participants’ stated priority of minimizing the added cost burden to families, particularly by reducing costs associated with the overall course of care following a child’s first primary care visit [21,36]. Meanwhile, the subtheme Provider Retention & Longevity aligns with enhancing clinician well-being. For example, in the adult PCMH, providers offload clinical work to a well-trained embedded PT, allowing each clinician to not only practice at the top of their license but also to optimize their time utilization [21]. It is reasonable to anticipate similar benefits in pediatrics with the implementation of an embedded pediatric PT.

To inform any potential pediatric implementation, there are multiple adult primary care physical therapy practice models that have emerged [14,37]. For example, Kaiser Permanente in Northern California incorporated PTs into the primary care clinic through shared clinic space and integrated electronic medical records with a streamlined referral system. A developed triage system at intake gives patients the choice of seeing the PT with the primary care physician, after seeing the physician, or before seeing the physician [21,37]. Similarly, the PCMH model has been used at community-based military hospitals, where PTs were provided a treatment room adjacent to the primary care providers [4]. In this model, patients with musculoskeletal complaints were given the option to see the PT before a primary care provider. Patient satisfaction scores were substantially higher for those seeing a PT in primary care versus referral to an outpatient clinic (96% vs. 74%); the service led to decreased utilization of imaging, no reports of patient harm, and cost savings, with 35% fewer individuals seeking out-of-network care [4]. Moving physical therapy upstream within the context of the healthcare system addresses needs around social determinants of health, prevention, early intervention, increased quality and efficiency of care, and decreased long-term complications [38].

Based on the current study findings, there appears to be an untapped opportunity to embed pediatric PTs in the pediatric PCMH, with PTs contributing significantly to value-based health care. However, limitations of this study must be noted. For one, the results represent a sample based on convenience, comprising pediatricians geographically located only in western Oregon. Pediatricians were recruited from both rural and urban, hospital-based and private settings, and recruitment continued until thematic analysis revealed saturation of ideas and themes. Nonetheless, the sampling methods here do limit the generalizability of results. Additionally, it is acknowledged in the literature that thematic analysis is susceptible to researcher bias, though trustworthiness has been assured here through the use of published methodology [23]. Despite these limitations, this is the first known study exploring the potential for embedded pediatric physical therapy in the pediatric primary care setting. The results here are a robust reflection of current pediatrician insights that can guide initial work toward a pilot implementation of such a service. The findings here can be leveraged in an implementation both to spark conversations around specific provider priorities and to guide dialogue with pediatricians on where to focus the initial work of the embedded PT in order to obtain the largest benefit for the patient population seen in a specific primary care locale.

## Figures and Tables

**Table 1:** Participant and practice characteristics (n = 9).

Characteristic	Mean (Min–Max) or Count
Age	50.0 (37–61)
Female	6
MD (vs. DO)	7
Any specialty certifications	5
Years in practice	20.9 (9–33)
Years in pediatrics	20.2 (6–33)
Familiarity with pediatric PT ^1^	
5—very familiar	4
4—moderately familiar	1
3—somewhat familiar	4
Familiarity with primary care PT ^1^	
3—somewhat familiar	1
2—slightly familiar	4
1—not at all familiar	4
Urban practice setting (vs. rural)	6
Hospital-based practice (vs. private)	4

MD = doctor of medicine; DO = doctor of osteopathic medicine; PT = physical therapy. ^1^ Scored on a 5-point Likert-style scale.

## Data Availability

Raw transcripts of complete qualitative interviews are not able to be made available, due to confidentiality of participating providers in this study, as per institutional IRB approval. Limited material can be made available to interested researchers upon request.

## Appendix A. Semi-Structured Interview Schedule

Domain of Inquiry

Pediatrician/pediatric physical therapist (PT) perspectives on the potential of having a pediatric PT embedded in pediatric primary care medical home, similar to adult primary care PT (PCPT) implementation—particularly related to meeting the *Quadruple Aim* and getting the *right patient* the *right care* at the *right time*, amidst the realities of current pediatric healthcare practice.

Categories of the Topic

- Enhancing provider experience (per *Quadruple Aim*)
- Improving patient experience and clinical outcomes (per *Quadruple Aim*)
- Identified priorities in their clinic and community
- Clinicians’ current knowledge and utilization of pediatric PT
- Clinician initial insights into the potential of implementing pediatric PCPT

Question Stems

- Let’s start with the Quadruple Aim. [show it on page 2; briefly familiarize with it, PRN] We have found that when implementing PCPT services with our adult provider partners, *Enhancing Provider Experience* is a great place to start. Talk to me/us first about anything that would **enhance your experience and satisfaction** in your work.
  - In what way…? Tell me about that…? Such as…?
  - Anything else?
- Alright, now talk to me/us about **your top challenges** in your work related to any and all aspects of providing care for children and families.
  - In what way…? Tell me about that…? Such as…?
  - Anything else?
- What are some **priority** types of patients, conditions, and/or diagnoses that you feel are **most difficult** to manage well?
  - How so? Talk to me/us [*more*] about **specific challenges** you have in working with a couple of those types of patients/conditions.
- Thinking about your **sense of competence and effectiveness** in working with a range of diagnoses, what *personal traits and behaviors* are important?
  - In what way…? Tell me about that…? Such as…?

*Alright, now I/we would like to explore the potential of having a pediatric physical therapist embedded in the pediatrician’s office. I/we want to start with an introductory **video** discussing primary care physical therapy.*

- First, do you have any questions? [answer these; any resulting conversation is welcomed]
- Having had this brief introduction to the potential of PTs practicing side-by-side with providers in primary care, what are your **initial thoughts**?
  - In what way…? Tell me about that…? Such as…?
- How might having a pediatric PT available in the clinic **enhance your work** and/or **better support patient care**?
  - In what way…? Tell me about that…? Such as…?
  - Anything else?
  - Using the words of our partners in adult primary care PT practices, what might PT be able to “*take off the provider’s plate*”?
- What **challenges** do you see around implementing a pediatric primary care PT service? These could be related to office culture, providers working side-by-side with a PT, logistics, or anything.
  - In what way…? Tell me about that…? Such as…?
  - Anything else?
- In adult primary care where PTs consult side-by-side with the provider in the exam room and/or do warm-handoffs, what types of patients/families/diagnoses could you see providers collaborating in this way with pediatric PTs?
  - In what way…? Tell me about that…? Such as…?
  - [Seek specifics as needed.]
- Wrapping up, this is really about trained PTs working upstream alongside providers to **add value**. Summarize for me/us your thoughts related to the **costs and benefits** (i.e., the “value proposition”) of potentially implementing a pediatric primary care PT service like we’ve discussed today.
- **Anything else** that you would like to add?

## Appendix B. Research Study Description and Participant Characteristics

You are being asked to participate in an audio-recorded interview session. The purpose is to gain your thoughts around the potential benefits and challenges of embedding a PT in the pediatrician’s office. This appears to have never been done before in pediatric primary care practice, though it is happening in general adult primary care. All recorded comments will be transcribed and analyzed—your comments will not be connected to your name, and all data will be protected per research standards. The audio recordings will be deleted once transcribed. We plan to publish our study findings and use them to help implement a pilot program for embedded pediatric primary care PT in Oregon. There are no anticipated benefits directly for you, and risk is minimal. Your participation is totally voluntary, and you can stop at any time. By continuing with this form and interview, you consent to participate in this research study.

**Participant Data:**

Unique ID: __________

Age in years: ___________ Gender: male/female/other: ______________________

Profession: MD/PA/NP/PT Credentials (e.g., PT, DPT, PCS): ___________________________

Years in *pediatric* practice: ___________ Years *total* practicing in your profession: ___________

List any specialties/certifications: _______________________________________________________________

Which of the following patient age groups do you see **most regularly** in your practice (*circle **top 1 or 2***)?

- Infants & Toddlers
- Preschoolers
- Elementary Age
- Middle/High School Age

In which of the following categories does **a good amount** of your caseload tend to fall (*circle **top 1 or 2***)?

- Musculoskeletal
- Developmental Disability/Long-term Conditions
- Metabolic/Lifestyle
- Mental Health
- General Medical (e.g., organ systems, illness)
- Other: _________________________________________________

How familiar are you with **pediatric physical therapy**, including what pediatric PTs can offer in care for children and families, as well as their scope of practice?

1—Not at all familiar

2—Slightly familiar

3—Somewhat familiar

4—Moderately familiar

5—Very familiar

How familiar are you with **primary care physical therapy**, including what services PTs can offer in primary care?

1—Not at all familiar

2—Slightly familiar

3—Somewhat familiar

4—Moderately familiar

5—Very familiar
